# Time-trends and predictors of interhospital transfers and 30-day rehospitalizations after acute coronary syndrome from 2000-2015

**DOI:** 10.1371/journal.pone.0255134

**Published:** 2021-07-22

**Authors:** J. Afonso Rocha, José Carlos Cardoso, Alberto Freitas, Thomas G. Allison, Luís F. Azevedo

**Affiliations:** 1 Cardiovascular Rehabilitation Unit, Department of Physical Medicine and Rehabilitation, Centro Hospitalar Universitário São João, Porto, Portugal; 2 Center for Health Technology and Services Research (CINTESIS), Faculty of Medicine, University of Porto, Porto, Portugal; 3 Department of Cardiology, Centro Hospitalar Universitário São João, Porto, Portugal; 4 Faculty of Medicine, University of Porto, Porto, Portugal; 5 Department of Community Medicine, Information and Health Decision Sciences (MEDCIDS) and Center for Health Technology and Services Research (CINTESIS), Faculty of Medicine, University of Porto, Porto, Portugal; 6 Mayo School of Medicine and Science, Rochester, Minnesota, United States of America; Azienda Ospedaliero Universitaria Careggi, ITALY

## Abstract

**Aims:**

Assess trends and factors associated with interhospital transfers (IHT) and 30-day acute coronary syndrome (ACS) rehospitalizations in a national administrative database of patients admitted with an ACS between 2000–2015.

**Methods and results:**

Cohort study of patients hospitalized with ACS from 2000 to 2015, using a validated linkage algorithm to identify and link patient-level sequential hospitalizations occurring within 30 days from first admission (considering all hospitalizations within the 30-day timeframe as belonging to the same ACS episode of care-ACS-EC). From 212,481 ACS-EC, 42,670 (20.1%) had more than one hospitalization. ACS-EC hospitalization rates decreased throughout the study period (2000: 207.7/100.000 person-years to 2015: 185,8/100,000 person-years, p for trend <0.05). Proportion of IHT increased from 10.5% in 2000 to 20.1% in 2015 compared to a reduction in both planned and unplanned 30-day ACS rehospitalization from 9.0% in 2000 to 2.7% in 2015. After adjusting for patient and first admission hospital’s characteristics, compared to 2000–2003, in 2012–2015 the odds of IHT increased by 3.81 (95%CI: 3.65–3.98); the odds of unplanned and planned 30-day ACS rehospitalization decreased by 0.36 (95%CI: 0.33; 0.39) and 0.47 (95%CI: 0.43; 0.53), respectively. Female sex, older age and the presence and severity of comorbidities were associated with lower likelihood of being transferred or having a planned 30-day ACS rehospitalization. Unplanned 30-day ACS rehospitalization was more likely in patients with higher comorbidity burden.

**Conclusion:**

IHT and 30-day ACS rehospitalization reflect coronary referral network efficiency and access to specialized treatment. Identifying factors associated with higher likelihood of IHT and 30-day ACS rehospitalization may allow heightened surveillance and interventions to reduce rehospitalizations and inequities in access to specialized treatment.

## Introduction

Timely access to specialized care, namely coronary angiography and revascularization, are crucial for survival and prognosis in patients hospitalized with acute coronary syndrome (ACS) [1]. Simultaneously, in-hospital survival after ACS has dramatically increased with a higher proportion of patients being discharge alive from hospital who are at risk for early rehospitalization, worse outcomes and increased healthcare costs [2].

Interhospital transfers (IHT) of patients admitted with ACS are common since experienced specialized catheterization laboratories are not universally available among hospitals. In the Prague study, from 300 patients presenting to hospital within 6-hours of acute myocardial infarction (MI), those who were randomized to immediate transport for primary angioplasty were less likely to die or being readmitted in the first 30-days due to reinfarction or stroke [3, 4]. However, concerns remain on the real beneficial impact of IHT on outcomes since transferred patients have a lower risk factor profile and comorbidity burden compared to non-transferred patients [5].

Early rehospitalizations after hospital discharge for ACS, albeit not all preventable, are a marker for poor quality of care and efficiency of both in-hospital and post-discharge care, and increasingly considered a quality performance measure and a driver of healthcare costs in different medical and surgical conditions [6]. Thereby, there has been a considerable interest from hospitals, clinicians and policymakers to identify, understand and improve factors associated with 30-day rehospitalizations, especially those having ACS as principal diagnosis. Several studies have inconsistently reported different predictors for rehospitalization after MI with overall small effect sizes on the probability of 30-day rehospitalization [7]. The strongest predictors reported for 30-day ACS rehospitalization were older age, heart failure and previous MI [2, 8]. Nonetheless, little is known on rehospitalization rates and predictors of 30-day ACS rehospitalization after implementation of primary percutaneous coronary intervention (PCI) for ST-segment elevation MI (STEMI) and regarding other ACS subtypes.

Administrative health data, namely hospital discharge data, uses hospitalization as the unit of observation and is seldomly accessible to researchers with patient-level unique identifiers allowing for detection of sequential hospitalizations. Considering the country-level and regional-level heterogeneity in coronary referral networks to PCI-capable centers for management of MI patients and in the coding practices among institutions, significant distortions arise when estimating MI hospitalizations rates, proportion of patients submitted to revascularization procedures and in-hospital mortality [9]. Thereby, identifying patient-level sequential hospitalizations with an ACS-related code as principal diagnosis, and using the 30-day time-frame to group them as the same episode of care, might allow to minimize these distortions [9, 10].

Our primary aim was to describe long-term trends (2000–2015) in hospitalization for ACS, rates of IHT and, among patients surviving hospitalization for ACS, rates of 30-day rehospitalization having ACS as principal diagnosis, using a nationwide hospital discharge administrative database, and considering a 30-day ACS episode of care (ACS-EC) as the unit of observation. Our secondary aim was to describe patient characteristics, clinical factors and comorbidities, accounting for admitting hospital PCI-capability and size, associated with an increased likelihood for IHT and 30-day ACS rehospitalization.

## Methods

We performed a retrospective study using data from the administrative national hospital discharge database provided by the Portuguese Ministry of Health’s Central Administration for the Health System (ACSS) which includes hospitalizations occurring in all public acute care hospitals of the Portuguese Public National Health Service in mainland Portugal. To assure accuracy and coherence of coding procedures we restricted our analysis to all consecutive hospitalizations, in patients older than 30 years, occurring between 2000 and 2015, since from 2016 onwards there was a gradual and heterogenous transition from the International Classification of Diseases - 9th revision—clinical modification (ICD9-CM) to the ICD10. Hospital’s case-mix index (CMI) was obtained from the annual report by ACSS [11]. The case-mix index is the average relative Diagnostic Related Groups-DRG weight of a hospital’s inpatient discharges, calculated by summing the severity of the DRG weight for each discharge and dividing the total by the number of discharges. The CMI reflects the diversity, clinical complexity, and resource needs of all the patients in the hospital. A higher CMI indicates a more complex and resource-intensive case load [12].

Data were provided by the ACSS, an official organ of the Ministry of Health, through a cooperation protocol with the Faculty of Medicine, University of Porto, and CINTESIS (Center for Health Technology and Services Research), for research purposes. The dataset used in this study, which includes administrative records in a pre-anonymized form, and its use was approved by the ACSS for research purposes. No primary data were collected as part of the present study. Being a previously anonymized secondary dataset, no informed consent or ethical approval was pursued. All data were analyzed under data security and privacy policies. Access to data can be obtained, through collaboration protocols upon request to ACSS (geral@acss.min-saude.pt).

### Study population

A preliminary exploratory analysis revealed that coding procedures for ACS hospitalizations varies considerably between institutions and with time, especially in the case of IHT or planned ACS rehospitalization for specialized care and treatment, ranging from both institutions (referring and receiving) coding the ACS hospitalization and the procedure to only the receiving institution coding the hospitalization episode and procedure either as an inpatient or outpatient code and using either ACS related codes (ICD9-CM codes 410.x, 411.0–411.1) or coding the episode using an 414.x code (other forms of chronic ischemic disease). Since our aims were to study patterns of interhospital transfers having ACS as principal diagnosis, to identify 30-day ACS readmissions and to group all sequential ACS hospitalizations occurring within a 30-day time frame (defined as ACS-episode of care-ACS-EC), we restricted our analysis to hospitalizations having only ACS-related diagnosis (410.x; 411.x; 414.x). We used standard ICD9-CM codes for cardiac procedures and the Charlson comorbidities algorithm proposed by Quan et al. [13] (S1 Table).

Since ICD9-CM does not have a specific coding allowing identification of ACS subtypes, we used codes 410.0–410.6 and 410.8, for STEMI, codes 410.7 and 410.9, for non–ST-segment elevation myocardial infarction (NSTEMI) and code 411.0–411.1 for unstable angina (UA) [14].

### Identification of patient-level sequential hospitalizations and definition of ACS episode of care (ACS-EC)

Administrative inpatient discharge data uses hospitalizations as their unit of observation, irrespective of whether they belong to the same patient and/or are part of a contiguous set of hospitalizations within the same ACS-EC. In order to identify and chronologically link sequential hospitalizations belonging to the same patient, and considering the lack of direct patient identifiers, we used a previously validated internal deterministic linkage method using indirect identifiers (gender, date of birth, residential code), which showed a sensitivity of 98.4 [95%CI: 98.4; 98.5]; specificity of 97.8 [95%CI: 97.6; 98.0] and Cohen’s kappa of 0.9 to identify same-patient sequential hospitalizations having ACS-related codes as principal diagnosis compared to using a pseudonymized unique patient identifier [15].

Within each matched-group, time (in days) between discharge date of first hospitalization and admission date of subsequent hospitalization was calculated and all hospitalizations occurring within 30 days were considered as pertaining to the same ACS-EC [9]. Considering the first pair of contiguous hospitalizations within each episode of care, we classified the second hospitalization in the pair as IHT (< = 1 day between hospitalizations), 30-day ACS rehospitalization (within 2–30 days from first hospital admission), or as a new ACS-EC (>30days from first hospital admission). Early rehospitalizations were further subdivided into planned or unplanned, according to a specified administrative code at hospital admission.

**Fig 1:**
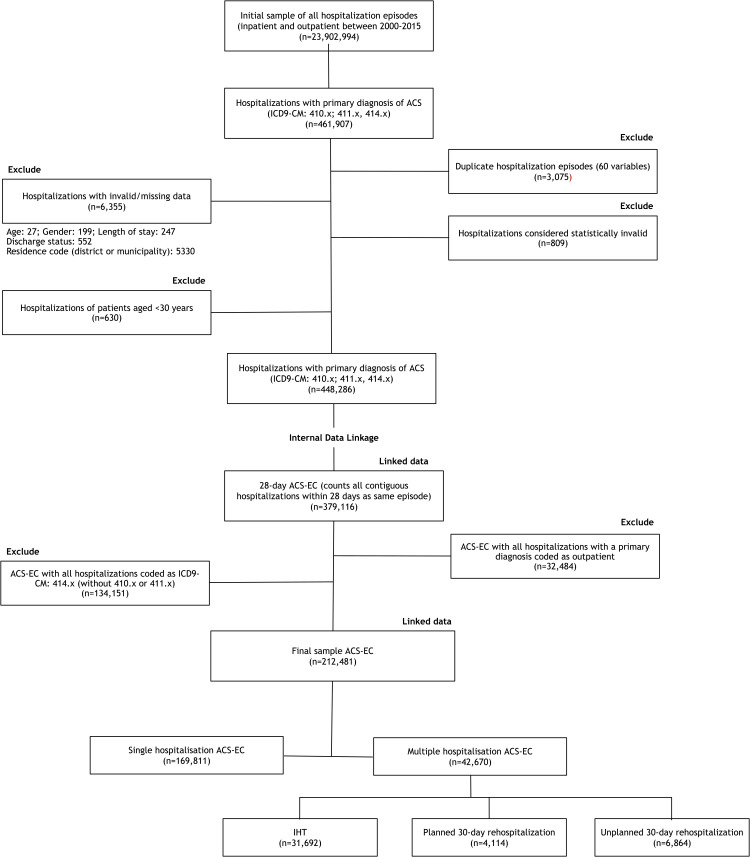
Flowchart of selection of ACS episodes of care.

Fig 1 shows the steps taken to select a final sample of patient-level linked hospitalizations. Considering we included both inpatient and outpatient codes and also 414.x codes, additional exclusion criteria were considered for episodes having only outpatient codes (n = 32,484) or 414.x codes (n = 134,151) in the sequence of hospitalizations, since it would be unlikely to be an ACS-related episode of care.

### Statistical analysis

Descriptive statistics are presented as mean and standard deviation (SD), for continuous variables, and percentages/proportions, for categorical variables. For the purpose of assessing time-trends in ACS hospitalizations, IHT and 30-day ACS rehospitalizations, we calculated the annual percent change (APC) by fitting a least squares regression line to the natural logarithm of the rates, using the calendar year as an independent (predictor) variable [16].

We derived age and sex standardized ACS-EC and hospitalization rates and their 95% confidence intervals using the Poisson approximation, following the direct standardization method proposed by Armitage and Berry [17, 18]. For standardization we used the Scandinavian Standard Population and, for country specific data, the results of the 2011 national survey (CENSUS 2011) [19], stratified by 5 year age groups and gender.

Identification of factors associated with the likelihood of being transferred to another hospital, being readmitted due to ACS within 30 days of first hospitalization, either planned or unplanned, compared to an ACS-EC with a single hospitalization, was evaluated using multinomial logistic regression (generalized logit model), with type of ACS-EC (single hospitalization, IHT, 30-day planned and unplanned ACS hospitalization) as dependent variable, adjusting for the characteristics at initial hospitalization and of first contact hospital, including PCI-capability, size and the average annual case-mix. The regression model was run only on those surviving first hospitalization (n = 192,558). Single hospitalization ACS-EC was chosen as reference category and the results are presented as OR with 95% confidence interval (95% CI) for each type of ACS-EC compared to single hospitalization ACS-EC.

Since our aim was to study factors, in first hospitalization, associated with odds of IHT or 30-day ACS rehospitalization, we adjusted our model to the PCI-capability of first-contact hospital, considering PCI-capable those institutions that had a full working catheterization laboratory (working 24 hours a day/7 days a week), taking into account changes in status and hospital merges occurring throughout the study period [20, 21].

To select variables for the multinomial logistic multivariable model we used a likelihood ratio test p-value <0.05. To build our multinomial multivariable model we used a backward stepwise approach with entry criteria p<0.05 and removal criteria p>0.10. Furthermore, we verified that remaining variable coefficients did not change by more than 20–25% with the reduced model. Results are presented as odds ratio (OR) with 95% CI, for each multiple hospitalization category compared to single hospitalization ACS-EC [22].

## Results

### Descriptive statistics: Annual time trends and sample characteristics

During the study period we identified 448,286 valid hospitalizations coded 410.x, 411.0–411.1 or 414.x, corresponding to 379,116 ACS-EC. Of these, 166,635 (43.9%) were excluded because all hospitalizations had only 414.x codes or only outpatient codes, leaving a final sample of 212,481 ACS-EC (Fig 1).

**Fig 2:**
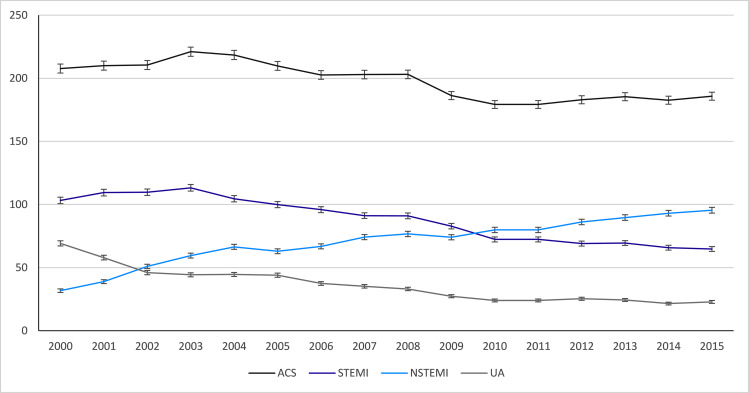
Age-sex standardized ACS-EC hospitalization rate between 2000–2015, per 100,000 person-years.

While the total number of ACS-EC did not change significantly throughout the study period (from 12,777 in 2000 to 12,999 in 2015), the age-sex standardized ACS-EC hospitalization rates decreased from 207,7/100,000 person-years, in 2000, to 185,8/100,000 person-years in 2015 (p for trend <0.05), with a marked decrease in the incidence of both STEMI and UA almost offset by an increase in NSTEMI ACS-EC (Fig 2).

In-hospital mortality occurred in 21,660 (10.2%) ACS-EC, increasing from 1,359 (10.6%) deaths/per year in 2000 to a maximum of 1,652 (11.6%) in 2003, steadily decreasing thereafter to a minimum of 1,059 (8.1%) in 2015. The decrease in mortality was more expressive for NSTEMI (15.6% in 2000 to 7.8% in 2015, average annual change -0.46%) than for STEMI (15.1% in 2000 to 10.4% in 2015; average annual change -0.29%). For those having only one hospitalization and patients transferred, between 2000 and 2015, in-hospital mortality decreased from 1,220 (11.9%) to 983 (9.8%) and from 92 (6.8%) to 58 (2.2%), respectively. For 30-day ACS rehospitalizations, there was a slight increase in in-hospital mortality from 44 (6.5%) to 16 (7.6%) and from 3 (0.6%) to 2 (1.0%), respectively for unplanned and planned 30-day ACS rehospitalization.

**Fig 3:**
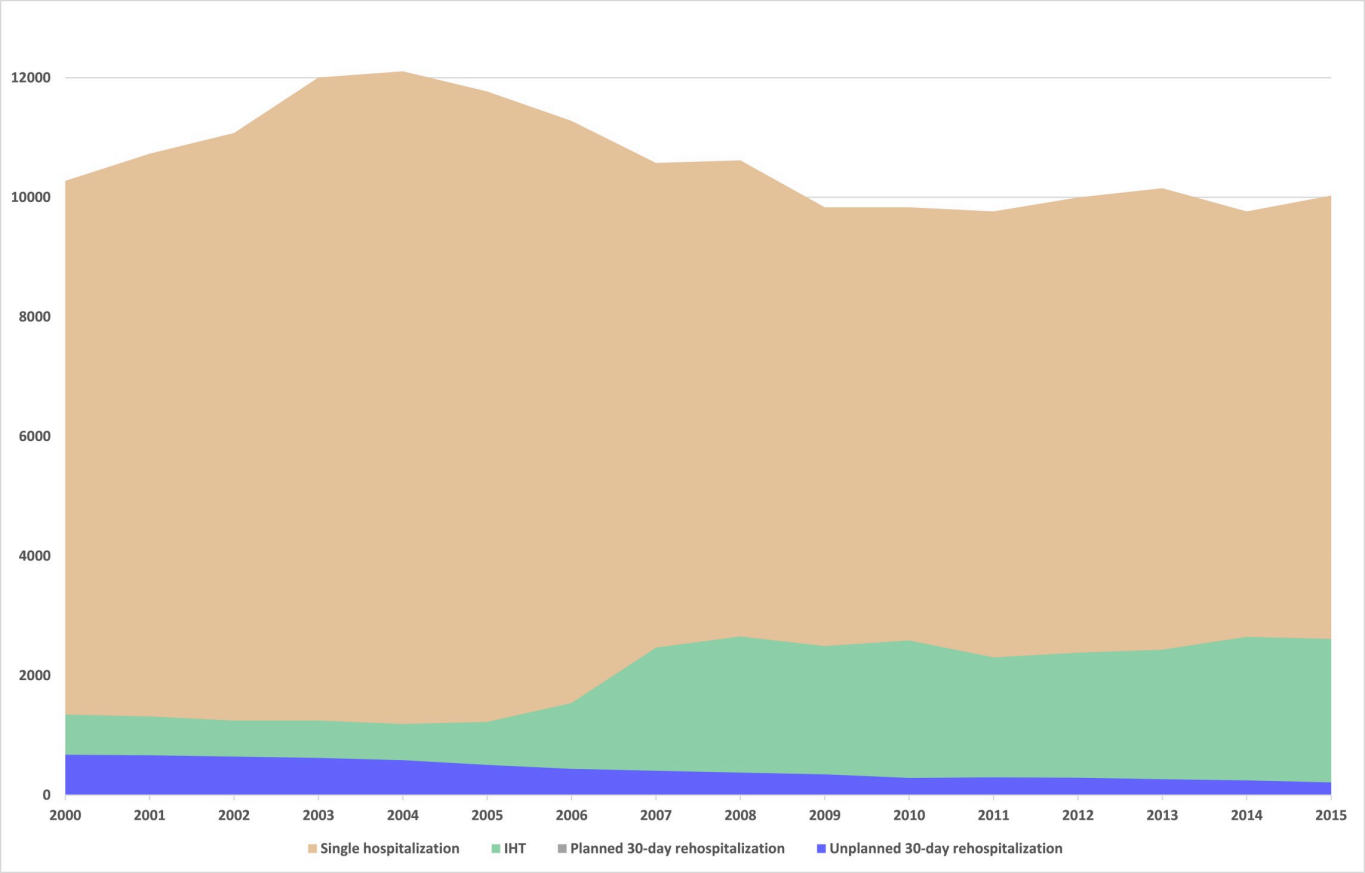
Number of ACS rehospitalizations, per year, for single hospitalization ACS-EC and for multiple hospitalizations ACS-EC.

Most ACS-EC had only one hospitalization (79.9%), while 31,692 (14.9%) were transferred to another hospital and 10,978 (5,2%) had an ACS rehospitalization within the first 30 days, with 6,864 (3.2%) being unplanned admissions (Fig 3). From 2000 to 2015, there was an increase in the proportion of ACS-EC having multiple hospitalizations driven by a steady increase in IHT from 10.5% in 2000 to 20.1% in 2015, resulting in an annual percent change of 2.2% (95%CI: 1.1–3.4). On the other hand, 30-day ACS rehospitalizations, planned or unplanned, decreased significantly between 2000 and 2015 from 9.0% to 2.7% with an annual percent change of -8.1 (95%CI: -8.6; -7.5). Of the 10,978 30-day ACS rehospitalizations the majority were unplanned readmissions (3.2%: 6,864 unplanned ACS rehospitalization/212,481 ACS episodes of care). Median time to 30-day ACS rehospitalization was 12 days [P50 (P25-P75): 12 (6–19)], being 12 (7–20) days for planned admissions and 11 (6–18) days for unplanned ACS rehospitalizations. Most ACS rehospitalization occurred in the first 14 days, with 33.8% occurring within the first 7 days, 27.8% between 8 and 14 days; 20.9% between 15 and 21 days and 17.5% occurring between 22 and 30 days after initial admission.

Mean age of patients increased steadily from 66.4 (12.5) years, in 2000, to 69.0 (13.7) years, in 2015, with the proportion of those having more than 80 years old increasing from 14.2% in 2000 to 26.0% in 2015. One third of ACS-EC occurred in women, remaining stable throughout the study period. Median length of stay decreased significantly from P50 (P25-P75): 8.0 (4.0–11.0) days in 2000 to 5.0 (3.0–9.0) days in 2015.

Aggregating data from all hospitalizations within an ACS-EC, we found that 120,503 (56.7%) were submitted to cardiac catheterization (2000: 33.8% to 2015: 72.1%), 71,719 (33.8%) to PCI (2000: 8.7% to 2015: 50.1%) and 10,050 (4.0%) to coronary artery bypass grafting (2000: 4.2% to 2015: 4.5). The proportion of those not submitted to any cardiac diagnostic or revascularization procedure between 2000 and 2015 decreased from 63.0% to 26.3%, respectively.

Of those patients having only one ACS hospitalization, 52,941 (31.2%) were submitted to PCI and 2,487 (1,5%) were submitted to CABG. Amongst patients transferred to another institution, 15,018 (47,4%) had a PCI, with 8,462 having the procedure during first hospitalization, 11,540 after being transferred and 1,734 submitted to PCI during subsequent hospitalizations.

Among patients having a 30-day ACS unplanned rehospitalization, 2,215 (32.2%) had a PCI and 632 (9,2%) were submitted to CABG. Within those having a 30-day ACS unplanned rehospitalization 1,295 had a PCI during the first hospitalization, 1,150 at the first 30-day unplanned ACS readmission and 344 had the procedure during subsequent hospitalizations.

Planned 30-day readmissions showed a PCI rate of 1,575 (38,3%) and CABG rate of 1,275 (31.0%). Of these, 664 had the PCI during first hospitalization, 1,248 during the first 30-day planned ACS readmission and 209 had the procedure during subsequent hospitalizations.

### Characteristics associated with number and type of hospitalizations within an ACS-EC

Table 1 describes patient and hospital characteristics comparing single hospitalization ACS-EC to IHT and 30-day planned and unplanned ACS rehospitalization.

**Table 1:** Baseline characteristics of ACS-EC and comparison between different types of ACS-EC, for each variable category linked data.

Characteristic, n (%)	Single hospitalization (n = 169,811)	IHT (n = 31,692)	Planned 30-day rehospitalization (n = 4,114)	Unplanned 30-day rehospitalization (n = 6,864)
**Sex**				
Male	106,555 (62.7)	22,528 (71.1)	3,222 (78.3)	4,4415 (64.3)
Female	63,256 (37.3)	9,164 (28.9)	892 (21.7)	2,449 (35.7)
**Age**				
Mean (SD)	68.9 (13.5)	65.4 (12.2)	63.5 (11.0)	69.5 (12.2)
Age > = 75 years	66,377 (39.1)	8,395 (26.5)	698 (17.0)	2,630 (38.3)
***10-year age groups***				
30 thru 39	2,975 (1.8)	579 (1.8)	76 (2.8)	74 (1.1)
40 thru 49	13,612 (8.0)	3,083 (9.7)	427 (10.4)	425 (6.2)
50 thru 59	26,661 (15.7)	6,395 (20.2)	941 (22.9)	922 (13.4)
60 thru 69	36,935 (21.8)	8,498 (26.8)	1,325 (32.2)	1,661 (24.2)
70 thru 79	48,197 (28.4)	9,224 (29.1)	1,122 (27.3)	2,250 (32.8)
80+	41,431 (24.4)	3,913 (12.3)	223 (5.4)	1,532 (22.3)
**Admission year**				
2000–2003	44,086 (26.0)	5,160 (16.3)	1,624 (39.5)	2,609 (38.0)
2004–2007	45,732 (26.9)	6,422 (20.3)	1,192 (29.0)	1,939 (28.2)
2008–2011	40,050 (23.6)	10,038 (31.7)	708 (17.2)	1,305 (19.0)
2012–2015	39,943 (23.5)	10,072 (31.8)	590 (14.3)	1,011 (14.7)
**Hospital characteristics**				
***Hospital size (beds)***				
< = 250	21,290 (12.5)	4,412 (13.9)	509 (12.4)	887 (12.9)
251–500	63,573 (37.4)	12,419 (39.2)	1,544 (37.5)	2,542 (37.0)
501–750	35,934 (21.2)	6,069 (19.1)	887 (21.6)	1,390 (20.3)
>750	49,014 (28.9)	8,792 (27.7)	1,174 (28.5)	2,045 (29.8)
***Intervention profile***				
Non-PCI capable	72,178 (42.5)	27,486 (86.7)	2,005 (48.7)	3,101 (45.2)
PCI-capable without open heart surgery	74,403 (43.8)	3,141 (9.9)	1,532 (37.2)	2,814 (41.0)
PCI-capable with open heart surgery	23,230 (13.7)	1,065 (3.4)	577 (14.0)	949 (13.8)
***Hospital case-mix***				
1^st^ tertile (< = 0.987)	46,051 (27.1)	15,113 (47.7)	1,306 (31.7)	1,943 (28.3)
2^nd^ tertile (>0.987; < = 1.15)	52,674 (31.0)	8,546 (27.0)	1,379 (33.5)	1,978 (28.8)
3^rd^ tertile (>1.15)	71.086 (41.9)	8,033 (25.3)	1,429 (34.7)	2,943 (42.9)
**ACS type on admission HE**						
STEMI	77,033 (45.4)	12,930 (43.2)	1,785 (46.1)	2,447 (41.7)
NSTEMI	61,337 (36.1)	12,052 (40.3)	1,089 (28.1)	2,114 (36.0)
UA	31,441 (18.5)	4,943 (16.5)	998 (25.8)	1,304 (22.2)
**Cardiac procedures (aggregated)**				
No procedure	77,528 (45.7)	2,318 (7.3)	286 (7.0)	2,140 (31.2)
Fibrinolysis	7,463 (4.4)	2,158 (6.8)	275 (6.7)	351 (5.1)
Coronary angiography	84,708 (49.9)	27,964 (88.2)	3,449 (83.8)	4,382 (63.8)
Coronary angioplasty	52,941 (31.2)	15,018 (47.4)	1,575 (38.3)	2,215 (32.3)
CABG	2,487 (1.5)	5,656 (17.8)	1,275 (31.0)	632 (9.2)
**Clinical severity indicators (first admission)**				
Cardiac arrest	4,093 (2.4)	319 (1.0)	19 (0.5)	31 (0.5)
	5,305 (3.1)	524 (1.7)	23 (0.6)	43 (0.6)
VFib./Vflutter	4,682 (2.8)	802 (2.5)	61 (1.5)	99 (1.4)
Afib/AFlutter	21,986 (12.9)	2,781 (8.8)	206 (5.0)	712 (10.4)
Death^†^	19,482 (11.5)	1,427 (4,5)	71 (1.7)	682 (9.9)
**Comorbidities (first admission)**				
Charlson index > = 3	15,797 (10.5)	2,392 (7.6)	221 (5.4)	942 (13.8)
Myocardial infarction	13,500 (8.0)	2,820 (8.9)	307 (7.5)	830 (12.1)
CHF	30,081 (17.7)	3,811 (12.0)	341 (8.3)	1,246 (18.2)
PVD	6,594 (3.9)	1,160 (3.7)	115 (2.8)	358 (5.2)
CVD	11,401 (6.7)	1,395 (4.4)	155 (3.8)	403 (5.9)
Dementia	2,178 (1.3)	69 (0.2)	6 (0.1)	65 (0.9)
Paraplegia	509 (0.3)	59 (0.2)	2 (0.0)	11 (0.2)
COPD	11,006 (6.5)	1,617 (5.1)	168 (4.1)	419 (6.1)
Rheumatic disease	1,023 (0.6)	199 (0.6)	11 (0.3)	31 (0.5)
Peptic ulcer disease	1,427 (0.8)	170 (0.5)	27 (0.7)	52 (0.8)
Liver disease	1,979 (1.2)	280 (0.9)	24 (0.6)	72 (1.0)
Renal disease	13,938 (8.2)	1,662 (5.2)	172 (4.2)	698 (10.2)
Malignancy	3,087 (1.8)	348 (1.1)	36 (0.9)	125 (1.8)
Diabetes	47,243 (27.8)	9,129 (28.8)	1,052 (25.6)	2,277 (33.2)
Hypertension	94,839 (55.8)	18,217 (57.5)	2,160 (52.5)	3,861 (56.3)
Dyslipidemia	57,154 (33.7)	12,258 (38.7)	1,519 (36.9)	2,129 (31.0)
Obesity	19,012 (11.2)	4,187 (13.2)	375 (9.1)	664 (9.7)
Smoking	34,343 (20.2)	7,504 (23.7)	823 (20.0)	1,032 (15.0)

^†^ For ACS-episodes of care having more than one hospitalization, death rates were calculated having those discharged alive after index ACS hospitalization as denominator.

ACS: acute coronary syndrome; Afib/AFlutter: atrial fibrillation/atrial flutter; CABG: coronary artery bypass grafting; CHF: chronic heart failure; COPD: chronic obstructive pulmonary disease; CVD: cerebrovascular disease; ER: early readmission; NSTEMI: non-ST-segment elevation myocardial infarction; PCI: percutaneous coronary intervention; PVD: peripheral vascular disease; STEMI: segment elevation myocardial infarction; UA: unstable angina; Vfib/Vflutter: ventricular fibrillation/ventricular flutter.

Patients transferred to another institution or having a planned 30-day ACS rehospitalization were younger and more often male compared to single hospitalization and unplanned 30-day ACS rehospitalization. On the other hand, the proportion of patients over 80 years being transferred or having a planned 30-day ACS rehospitalization were significantly lower than for those having a single hospitalization.

Patients subsequently transferred to another institution had a higher proportion of NSTEMI, while those readmitted within 30-days of first admission had a slightly higher proportion of unstable angina compared to single hospitalization. Transferred patients and those with a planned 30-day ACS rehospitalization more commonly underwent cardiac procedures (coronary angiography, PCI, or coronary artery bypass graft surgery). Of the entire sample, 49.3% were admitted to hospitals with only noninvasive capabilities, 38.5% to hospitals with interventional capability without open heart surgery and 12.2% to hospitals with interventional capability and open-heart surgery.

IHT were more common amongst patients admitted to smaller, non-PCI capable institutions with lower case-mix index. There was an inverse relation between the likelihood of IHT and the interventional capabilities of the admitting hospital; 26.2% of those admitted to non-PCI capable hospitals were transferred, compared to only 3.8% of those admitted to PCI-capable without open heart surgery, and 4.1% of those admitted to hospitals that performed open heart surgery.

Presence of cardiac arrest, ventricular tachycardia or cardiogenic shock at initial hospitalization and in-hospital death were more common in those having single hospitalization. In-hospital death occurred in 21,660 (10.2%) of ACS-EC, ranging from 19,482 (11.5%) in single hospitalization episodes of care, 1,427 (4.5%) amongst those being transferred to 682 (6.8%) in those with 30-day unplanned ACS rehospitalizations.

Patients either transferred or with a planned 30-day ACS rehospitalization were less likely to have more than 3 Charlson’s comorbidities, heart failure, stroke, COPD, renal or liver disease and malignancy, but transferred patients had a higher prevalence of hypertension, dyslipidemia, obesity and smoking.

### Factors associated with type of ACS-EC (multinominal logistic model)

Univariate analysis is presented in S2 Table. Multinomial logistic regression analysis, adjusted for the characteristics of first contact hospital, showed that older age and female sex were independently associated with a lower likelihood of IHT and 30-day planned rehospitalization, compared to single hospitalization ACS-EC (Table 2). The effect of age on the likelihood of IHT was most apparent for patients aged 75 years or older.

**Table 2:** Estimated odds ratios [95% confidence intervals] for the multivariable multinomial logistic model with single hospitalization ACS-EC as reference category and adjusted for first contact hospital characteristics (PCI capability, size and case-mix index).

Characteristic	IHT	Planned 30-day rehospitalization	Unplanned 30-day rehospitalization
	OR [IC 95%]	OR [IC 95%]	OR [IC 95%]
**Sex:** Female vs male	0.76 [0.74; 0.79]	0.57 [0.53; 0.62]	0.93 [0.88; 0.98]
**Age**: > = 75 yrs. vs <75 yrs.	0.61 [0.59; 0.64]	0.49[0.45; 0.53]	1.15 [1.09; 1.22]
**Year of admission**			
2000–2003	1.00 [Reference]	1.00 [Reference]	1.00 [Reference]
2004–2007	1.39 [1.33; 1.45]	0.76 [0.70; 0.82]	0.68 [0.64; 0.73]
2008–2011	3.38 [3.24; 3.53]	0.55 [0.50; 0.61]	0.50 [0.46; 0.54]
2012–2015	3.81 [3.65; 3.98]	0.47 [0.43; 0.53]	0.36 [0.33; 0.39]
**ACS type**			
STEMI	1.00 [Reference]	1.00 [Reference]	1.00 [Reference]
NSTEMI	1.04 [1.01; 1.07]	0.96 [0.89; 1.04]	1.10 [1.03; 1.17]
Unstable angina	0.88 [0.84; 0.91]	1.17 [1.08; 1.27]	1.03 [0.96; 1.11]
**Clinical severity indicators**			
Cardiogenic Shock	1.68 [1.48; 1.91]	0.79 [0.50; 1.25]	0.78 [0.56; 1.08]
VFib./Vflutter	1.15 [1.04; 1.26]	0.74 [0.57; 0.97]	0.80 [0.65; 0.99]
Afib/AFlutter	0.75 [0.72; 0.79]	0.58 [0.50; 0.67]	0.84 [0.77; 0.92]
**Comorbidities**			
Charlson Index Score > = 3	0.81 [0.71; 0.92]	1.28 [0.97; 1.76]	1.21 [1.02; 1.44]
Myocardial infarction	0.97 [0.91; 1.02]	0.94 [0.83; 1.07]	1.40 [1.28; 1.52]
CHF	0.81 [0.77; 0.84]	0.72 [0.64; 0.82]	1.12 [1.04; 1.21]
PVD	0.91 [0.84; 1.00]	0.97 [0.80; 1.19]	1.46 [1.29; 1.66]
CVD	0.80 [0.75; 0.86]	0.84 [0.71; 1.00]	0.95 [0.85; 1.07]
Dementia	0.21 [0.16; 0.27]	0.32 [0.14; 0.71]	0.91 [0.70; 1.19]
Paraplegia	0.85 [0.62; 1.17]	0.15 [0.02; 1.04]	0.76 [0.42; 1.39]
COPD	0.84 [0.79; 0.90]	0.87 [0.74; 1.02]	0.96 [0.86; 1.08]
Peptic ulcer disease	0.75 [0.62; 0.90]	0.87 [0.59; 1.28]	0.88 [0.65; 1.19]
Renal disease	0.76 [0.71; 0.81]	0.84 [0.70; 1.00]	1.37 [1.24; 1.51]
Malignancy	0.72 [0.63; 0.82]	0.68 [0.48; 0.96]	1.05 [0.86; 1.28]
Diabetes	1.12 [1.09; 1.16]	1.09 [1.01; 1.17]	1.33 [1.26; 1.41]
Hypertension	1.11 [1.08; 1.14]	1.03 [0.96; 1.10]	1.03 [0.97; 1.09]
Dyslipidemia	1.04 [1.01; 1.07]	1.17 [1.09; 1.25]	0.94 [0.88; 1.00]
Smoking	1.03 [0.99; 1.06]	0.77 [0.70; 0.84]	0.82 [0.76; 0.88]

ACS: acute coronary syndrome; Afib/AFlutter: atrial fibrillation/atrial flutter; CABG: coronary artery bypass grafting; CHF: chronic heart failure; COPD: chronic obstructive pulmonary disease; CVD: cerebrovascular disease; ER: early readmission; NSTEMI: non-ST-segment elevation myocardial infarction; PCI: percutaneous coronary intervention; PVD: peripheral vascular disease; STEMI: segment elevation myocardial infarction; UA: unstable angina; Vfib/Vflutter: ventricular fibrillation/ventricular flutter.

The odds of being transferred has increased by more than 3-fold from 2000 to 2015, the likelihood or being readmitted within the first 30-days after ACS significantly decreased in the same time period. Compared to those admitted with STEMI, NSTEMI patients were more commonly transferred or have an unplanned 30-day rehospitalization, whereas those with UA were more likely to have a planned 30-day rehospitalization. Also, cardiogenic shock or ventricular fibrillation at presentation increased the odds of IHT, while presence of either ventricular or atrial fibrillation decreased the odds of 30-day rehospitalization.

The presence and severity of the comorbidities independently decreased the likelihood of IHT and, to a lesser extent, of planned 30-day rehospitalization. On the other hand, unplanned 30-day rehospitalization was more likely in those having a previous history of MI, chronic heart failure, peripheral vascular disease, chronic kidney disease and diabetes. Presence of cardiovascular risk factors such as hypertension, diabetes and dyslipidemia increased the likelihood of being transferred after admission for ACS, while only diabetes increased the odds for unplanned hospital rehospitalization.

## Discussion

Use of a nationwide administrative hospital discharge database for identification of all hospital admissions between 2000–2015 having acute coronary syndrome as principal diagnosis, linked at patient-level by a validated linkage algorithm to identify sequential hospitalizations, allowed us to study trends in age-sex adjusted incidence of ACS, assess the proportion and predictors of IHT and 30-day rehospitalization after an ACS. We found a steady decrease in age-sex standardized hospitalization rates for ACS with an increasing proportion of patients aged 75 or older and with a higher prevalence and severity of comorbidities. The number of ACS-EC having multiple hospitalizations increased by an average of 2.2% per year, due to higher IHT rate, while 30-day rehospitalizations, both planned and unplanned, decreased steeply throughout the study period. Furthermore, in-hospital mortality has decreased over time for those having one or multiple ACS rehospitalizations with the exception of in 30-day unplanned readmissions where it slightly increased.

Our findings suggest that, irrespective of first contact hospital characteristics, the likelihood of being transferred to another institution is higher between males, younger patients, those presenting with cardiogenic chock or ventricular fibrillation and patients having a lower prevalence and severity of comorbidities. Furthermore, unplanned 30-day rehospitalization was more common amongst those aged > = 75 years, males, admitted due to NSTEMI and patients with a previous history of MI, diabetes, chronic heart failure, peripheral vascular disease and chronic kidney disease.

### Temporal tends in ACS hospitalization incidence and in-hospital mortality

The decrease in age- and sex-adjusted hospitalization rates for ACS, STEMI and unstable angina, and the relative increase in NSTEMI, were in line with previous observations [14, 23, 24]. Yeh et al. [14], in a 10-year population-based study of more than 45,000 patients hospitalized due to ACS, found a step decline in STEMI age- and sex-adjusted incidence from 133/100,000 person-years in 2000 to 50/100,000 person-years in 2008. These declines in total ACS and STEMI incidence most likely reflect improvements in preventive measures, diagnostic accuracy and sensitivity and therapeutic strategies [25]. On the other hand, we also found an increase in NSTEMI incidence throughout the study period, especially between 2001–2004 and 2011–2015. These findings might be related to the introduction of troponin assays and change in their sensitivity overtime. Troponin assays were first included in MI definition in 2000 and introduced in clinical practice in Portugal at around the same time [26]. High-sensitivity became widely available in 2010 allowing for early detection of even very mildly increased troponin from localized myocardial necrosis, increasing NSTEMI incidence but also improving prognosis by allowing for earlier revascularization, earlier transfer to the coronary care unit, and earlier initiation of evidence-based treatments [27]. In fact, one of the biggest changes we observed was a dramatic increase in the use of interventional procedures. From 2000–2015 there was increased from 33.8% to 72.1% in cardiac catheterization with a five-fold increase in the proportion of patients submitted to PCI.

We, like others, found a steady decrease in-hospital mortality from ACS throughout the study period [28], more expressively for NSTEMI [14, 23]. The reasons for these trends are possibly complex and multifactorial. On one hand, introduction of more sensitive biomarkers, such as high sensitivity troponins, could have resulted in increased detection of less severe MI allowing for earlier treatment and better outcomes. On the other hand, the observation that mortality reduction is also present in STEMI hints for a role of improved management of MI, namely through coronary referral networks allowing for early identification of STEMI and high-risk NSTEMI patients, direct transportation for PCI-capable centers and, when not possible, early interhospital transfer for primary percutaneous intervention [29, 30]. Moreover, transferred patients have been shown to have a survival benefit compared to non-transferred patients [1, 31]. Ranasinghe et al., in a matched- cohort follow-up study comparing transferred to non-transferred patients, found those transferred had 40% less chance of dying in the first 30 days after an MI than non-transferred MI patients, irrespective of age, MI type and comorbidity burden [31]. Additionally, a survival benefit stemming from increasing rates of primary or early percutaneous coronary intervention, especially amongst those directly admitted to PCI-capable centers or transferred early after admission to non-PCI centers, are also likely to contribute to the lower mortality found in these patient groups.

### Temporal trends in IHT and 30-day rehospitalizations

In our study, due to the methodological approach used for linkage of records and identification of patient-level sequential hospitalizations, we only considered rehospitalizations coded as acute coronary syndrome. Nonetheless, the rate of unplanned 30-day rehospitalization for ACS found in our study was similar to other studies. A prospective cohort study of 3,387 patients admitted with MI in one of 53 different hospitals in China reported a total of 4.9% rehospitalization for a cardiovascular diagnosis, with a 3.3% rate of unplanned admissions due to angina, myocardial infarction or for revascularization procedures [32]. Similarly, in a retrospective analysis of a Medicare registry including 53,471 patients aged ≥65 years discharged home alive after an MI, the rate of unplanned 30-day rehospitalization having an admission diagnosis similar to index hospitalization was 3.2% [33].

Although not all hospital rehospitalizations can be prevented, 30-day rehospitalization is considered a marker for poor quality of care and inadequate coordination of post-discharge care, resulting in a significant burden to patients, caregivers and the healthcare system [19, 34]. Several studies have also found a steady decline in rates of rehospitalizations after MI [24, 35]. Chen et al., in a retrospective population-based study of 4,810 patients discharged alive after a first MI between 2001 and 2011, found a decline in 30-day rehospitalization rate from 20.5% in 2001–2003 to 15.8% in 2009–2011 and 36% lower odds of being readmitted within the first 30-days in 2009–2011 compared to 2001–2003 [35]. In our study, since we considered only rehospitalizations having ACS as principal diagnosis, we found an even more significant decrease in the odds of 30-day rehospitalization, with a reduction of 53% in 2012–2015 compared to 2000–2003, for planned rehospitalizations, and a reduction of 64% in 2012–2015 compared to 2000–2003, for unplanned rehospitalizations, even after adjustment for potentially confounding variables.

Dhamarajan K et al., in a retrospective study of Medicare fee-for-service claims data regarding 30-day rehospitalization between 2007–2009, found that 10% of patients were readmitted for the same condition after their index MI admission [36]. In a post-hoc analysis of the pexelizumab in MI trial involving 5,745 patients admitted with STEMI in 17 different countries, including 13 European countries, from 2004 to 2006, found an overall 30-day rehospitalization rate of 10.5% with Portugal having a lower rate of 7.0% [37]. Our results showed a lower rehospitalization rate of 5.2%, decreasing from 7.9% in 2000–2003 to 3.1% in 2012–2015. These differences can stem from the different methods used to identify and link sequential hospitalizations at the patient-level, to the restriction of rehospitalizations to those having the same codes as the first hospitalization and to the larger time-period including recent years where 30-day rehospitalization rates have been consistently declining.

### Predictors of IHT and 30-day rehospitalizations

There has been a growing interest, from healthcare providers and policy makers, to better understand national and regional-level trends in and predictors of IHT and early rehospitalizations. However, few studies provide information about predictors of IHT and 30-day rehospitalization after MI and no statistical models or risk scores are available to predict an individual’s risk of being either transferred or readmitted after discharge for MI [2, 38].

Receiving timely and specialized care for ACS, especially primary PCI, is a major determinant of cardiovascular outcomes. Nonetheless, since not all hospitals are capable of providing such specialized care, especially in rural areas, structured referral networks have to be in place to shorten time to access experienced specialized centers. We, like others [31, 39], found that, irrespective of acute coronary syndrome type, those transferred were more likely to have been admitted to a non-PCI capable hospital, be transferred for coronary angiography and/or revascularization and less likely to die. Ranasinghe et al. undertook a retrospective cohort analysis of >40,000 MI patients admitted to one of 161 hospitals in Australia, between 2004 and 2008, and found that, independently from age, type of MI and type of referring hospital, those transferred were also more likely to receive PCI and less likely to die in hospital, at 30 days and at 1 year follow-up [31]. This effect on mortality was consistent even after propensity score matching to overcome confounding by indication (those transferred are younger, have better risk factor profile and lower comorbidity burden) [31].

Rates of 30-day rehospitalization have been included as a healthcare quality performance measure and several initiatives and financial incentives have been set in place for the development of risk stratification tools to allow identification of patients at higher risk for 30-day rehospitalization and for implementation and testing of post-discharge care models [34]. Previous cohort studies found that cardiogenic shock at presentation, length of stay, complications of angiography or revascularization and presence of comorbidities such as diabetes, chronic kidney disease, heart failure, COPD, anemia, heart failure were associated with an increased risk of 30-day rehospitalization [24, 35]. Our study observed similar results for those with chronic heart failure, diabetes and chronic kidney disease but also found increased odds of 30-day rehospitalization amongst older patients, NSTEMI and those with previous history of myocardial infarction and peripheral vascular disease. Although most of these factors are not modifiable, early identification of high-risk groups of vulnerable patients might allow timely implementation of discharge and post-discharge strategies to reduce odds for early rehospitalizations. From a system and organizational standpoint, safe transition from hospital to the community or post-discharge care facilities requires centering decisions on the patient and caregivers, mitigating boundaries between different levels of care [6]. Further studies are needed to examine and compare the effectiveness of different post-discharge transitions of care and models for integrating hospital, hospice, community and homecare in reducing rehospitalization in different disease settings.

### Strengths and limitations

The strengths of the present study include its large nationwide sample of hospitalizations due to ACS, wide time-frame of 16 years and use of a validated linkage method for patient-level sequencing of hospitalizations and identification of IHT, planned and unplanned 30-day rehospitalizations.

Several limitations have to be acknowledged in the interpretation of our findings. As the present study was based on administrative discharge data throughout a long time period, we cannot exclude the possibility of biases from changes in coding practices overtime and differences between hospitals coding procedures affected our results [40, 41]. Additionally, whether the patient was transferred to another institution or readmitted with a similar diagnosis to the first hospitalization relies on the accuracy of the second hospitalization primary diagnosis coding, which may vary according to provider opinion and hospital billing practices. There is also the potential for unmeasured confounders in our findings since administrative claims doesn’t provide detailed information on patient-associated characteristics such as education, income, professional status, psychosocial factors, adherence to post-discharge planning and patient preference.

## Conclusions

The results of this large observational study provide knowledge into trends in hospitalizations for ACS and rates of IHT and 30-day rehospitalizations, among patients who survived hospitalization for ACS between 2000–2015. The rise in IHT, associated with a higher rate of coronary revascularization and lower mortality, possibly reflects the implementation of coronary referral networks providing timely access to specialized services. On the other hand, rates of 30-day unplanned hospitalization, a widely accepted quality performance measure, shows an encouraging and consistent decline throughout the study period.

Understanding which factors influence timely IHT and 30-day rehospitalizations might help identify inequities in access to specialized care and treatment, those at higher risk for adverse outcomes and the development of patient-centered interventions for both in-hospital and post-discharge care.

## Supporting information

S1 Table List of ICD9-CM codes used for data retrieval. (DOCX)

S2 Table Univariate multinomial logistic model for initial hospitalization characteristics with four-level outcome variable as the dependent variable (outcome), and using single-hospitalization ACS-EC as reference category. (DOCX)
