# Supplementary material for: Time-trends and predictors of interhospital transfers and 30-day rehospitalizations after acute coronary syndrome from 2000-2015
Source: PLoS One. 2021 Jul 22;16(7):e0255134. doi: 10.1371/journal.pone.0255134 (PMC8297861; doi:10.1371/journal.pone.0255134)
Supplement: S1 Table — (DOCX) [file pone.0255134.s001.docx]

**S1 Table. List of ICD9-CM codes used for data retrieval**

| **Description** | **Code** |
| --- | --- |
| **Acute coronary syndrome**  Acute MI  Other acute and subacute forms of ischemic heart disease | 410x  411x |
| **Other forms of chronic ischemic heart disease** | 414.x |
| ACS subtypes  Transmural MI  Subendocardial MI  Nonspecified location MI  UA  **Subgroups**  STEMI (=Transmural MI)  NSTEMI  UA  UA. NSTEMI | 410.0, 410.1; 410.2; 410.3, 410.4, 410.5; 410.6; 410.8  410.7  410.9  411  410.0, 410.1; 410.2; 410.3, 410.4, 410.5; 410.6; 410.8  410.7 + 410.9  411  410.7 + 410.9 + 411 |
| **Severity indicators** |  |
| Cardiac arrest | 427.5 |
| Cardiogenic Shock | 785.5 |
| Atrial fibrillation/flutter  Ventricular fibrillation/flutter | 427.3, 427.31, 427.3  427.1, 427.4, 427.41; 427.42 |
| **Cardiac procedures** |  |
| Cardiac diagnostic catheterization | 37.21,37.22, 37.23 |
| PCI | 00.66, 36.03, 36.04; 36.06, 36.07, 36.09 |
| CABG | 36.10–36.17, 36.19 |
| **Charlson comorbidities** |  |
| Myocardial infarction | 410x; 411x |
| Congestive heart failure | 398.91, 402.01; 402.11; 402.91 404.01-404.03; 404.11; 404.13; 404.91; 404.93; 425.4–425.9; 428.0-428.4; 428.9; |
| Peripheral vascular disease | 093.0, 437.3, 440.x, 441.x, 443.1–443.9, 47.1, 557.1, 557.9, V43.4 |
| Cerebrovascular disease | 362.34, 430.x–438.x |
| Dementia | 290.x, 294.1, 331.2 |
| Hemiplegia or paraplegia | 334.1, 342.x, 343.x, 344.0– 344.6, 344.9 |
| Chronic pulmonary Disease | 416.8, 416.9, 490.x–505.x, 506.4, 508.1, 508.8 |
| Rheumatic disease | 446.5, 710.0–710.4, 714.0– 714.2, 714.8, 725.x |
| Peptic ulcer disease | 531.x–534.x |
| Liver disease | 070.22, 070.23, 070.32, 070.33, 070.44, 070.54, 070.6, 070.9, 570.x, 571.x, 573.3, 573.4, 573.8, 573.9, 456.0–456.2, 572.2–572.8, V42.7 |
| Diabetes | 250.0–250.3, 250.8, 250.9, 250.4–250.7 |
| Renal disease | 403.01, 403.11, 403.91, 404.02, 404.03, 404.12, 404.13, 404.92, 404.93, 582.x, 583.0–583.7, 585.x, 586.x, 588.0, V42.0, V45.1, V56.x |
| Any malignancy, including lymphoma and leukemia, except  malignant neoplasm of skin | 140.x–172.x, 174.x–195.8, 200.x–208.x, 238.6 |
| Metastatic solid tumor | 196.x–199.x |
| **Other cardiac risk factors** |  |
| Hypertension | 401.0; 401.1; 401.9, 402.0; 402.1; 402.9; 403.0; 403.1; 403.9; 404.0; 404.1; 404.9, 405.0; 405.1; 405.9 |
| Dyslipidemia | 272.0, 272.1, 272.2, 272.4 |
| Obesity | 278.0-278.4; 278.8; V85.3; V85.4 |
| Smoking | 305.1; 649.0; V15.82; E869.4 |

CABG: coronary artery bypass grafting; MI: myocardial infarction; NSTEMI: non-ST-segment elevation myocardial infarction; PCI: percutaneous coronary intervention; STEMI: segment elevation myocardial infarction; UA: unstable angina;
