# Supplementary material for: Time-trends and predictors of interhospital transfers and 30-day rehospitalizations after acute coronary syndrome from 2000-2015
Source: PLoS One. 2021 Jul 22;16(7):e0255134. doi: 10.1371/journal.pone.0255134 (PMC8297861; doi:10.1371/journal.pone.0255134)
Supplement: S2 Table — (DOCX) [file pone.0255134.s002.docx]

**S2 Table. Univariate multinomial logistic model for initial hospitalization characteristics with four-level outcome variable as the dependent variable (outcome), and using single-hospitalization ACS-EC as reference category (excluding 19,923 dead at initial hospitalization, final sample 192,558)**

| **Characteristic** | **IHT** |  | **Planned 30-day rehospitalization** |  | **Unplanned 30-day rehospitalization** |  |
| --- | --- | --- | --- | --- | --- | --- |
|  | OR [IC 95%] |  | OR [IC 95%] |  | OR [IC 95%] |  |
| **Sex:** Female vs male | 0.72 [0.70; 0.74] |  | 0.49 [0.46; 0.53] |  | 0.99 [0.94; 1.04] |  |
| **Age**: >= 75 yrs vs <75 yrs. | 0.64 [0.63; 0.66] |  | 0.37 [0.34; 0.40] |  | 1.12 [1.07; 1.18] |  |
| **Year of admission** |  |  |  |  |  |  |
| 2000-2003 | 1.00 [Reference] |  | 1.00 [Reference] |  | 1.00 [Reference] |  |
| 2004-2007 | 1.20 [1.16; 1.25] |  | 0.71 [0.66; 0.76] |  | 0.72 [0.67; 0.76] |  |
| 2008-2011 | 2.15 [2.07; 2.23] |  | 0.48 [0.44; 0.52] |  | 0.55 [0.51; 0.59] |  |
| 2012-2015 | 2.14 [2.06; 2.22] |  | 0.39 [0.36; 0.43] |  | 0.42 [0.39; 0.45] |  |
| ***Hospital size (beds)*** |  |  |  |  |  |  |
| 251-500 (vs <=250) | 1.16 [1.11; 1.20] |  | 1.00 [0.90; 1.11] |  | 1.00 [0.92; 1.08] |  |
| 501-750 (vs <=250) | 1.09 [1.06; 1.12] |  | 1.01 [0.94; 1,10] |  | 0.96 [0.90; 1.02] |  |
| >750 (vs <=250) | 0.94 [0.91; 0.98] |  | 1.03 [0.94; 1.13] |  | 0.93 [0.87; 0.99] |  |
| **Hospital PCI capability** |  |  |  |  |  |  |
| PCI-capable without open heart surgery vs non-PCI capable | 0.11 [0.10; 0.11] |  | 0.74 [0.69; 0.79] |  | 0.95 [0.88;1.02] |  |
| PCI-capable with open heart surgery vs non-PCI-capable | 0.12 [0.11; 0.13] |  | 0.89 [0.81; 0.98] |  | 0.88 [0.84; 0.93] |  |
| ***Hospital case-mix*** |  |  |  |  |  |  |
| 1^st^ tertile (<= 0.987) | 1.00 [Reference] |  | 1.00 [Reference] |  | 1.00 [Reference] |  |
| 2^nd^ tertile (>0.987; <=1.15) | 0.49 [0.48; 0.51] |  | 0.92 [0.86; 1.00] |  | 0.89 [0.84; 0.95] |  |
| 3^rd^ tertile (>1.15) | 0.34 [0.33; 0.36] |  | 0.71 [0.66; 0.77] |  | 0.98 [0.93;1.04] |  |
| **ACS type** |  |  |  |  |  |  |
| STEMI | 1.00 [Reference] |  | 1.00 [Reference] |  | 1.00 [Reference] |  |
| NSTEMI | 1.14 [1.11; 1.17] |  | 0.74 [0.68; 0.79] |  | 1.05 [0.99; 1.11] |  |
| UA | 0.84 [0.81; 0.87] |  | 1.22 [1.12; 1.31] |  | 1.16 [1.08; 1.24] |  |

| **Clinical severity indicators** |  |  |  |  |  |
| --- | --- | --- | --- | --- | --- |
| Cardiac arrest | 1.08 [0.93; 1.24] |  | 0.61 [0.38; 0.99] |  | 0.62 [0.43; 0.90] |
| Cardiogenic shock | 1.42 [1.27; 1.58] |  | 0.48 [0.31; 0.76] |  | 0.60 [0.43; 0.82] |
| VFib | 1.05 [0.96; 1.13] |  | 0.62 [0.47; 0.80] |  | 0.63 [0.51; 0.77] |
| AFib | 0.68 [0.65; 0.71] |  | 0.38 [0.33; 0.43] |  | 0.83 [0.77; 0.90] |
| **Comorbidities** |  |  |  |  |  |
| Charlson index >=3 | 0.71 [0.67; 0.74] |  | 0.49 [0.42; 0.56] |  | 1.36 [1.27; 1.46] |
| Myocardial infarction | 1.10 [1.05; 1.15] |  | 0.91 [0.81; 1.03] |  | 1.55 [1.44; 1.68] |
| CHF | 0.71 [0.68; 0.73] |  | 0.47 [0.42; 0.53] |  | 1.17 [1.09; 1.24] |
| PVD | 1.03 [0.96; 1.09] |  | 0.77 [0.64; 0.93] |  | 1.47 [1.32; 1.64] |
| CVD | 0.72 [0.68; 0.76] |  | 0.61 [0.52; 0.72] |  | 0.99 [0.89; 1.10] |
| COPD | 0.79 [0.75; 0.84] |  | 0.62 [0.53; 0.72] |  | 0.95 [0.86; 1.06] |
| Rheumatic disease | 1.06 [0.91; 1.23] |  | 0.45 [0.25; 0.81] |  | 0.76 [0.53; 1.09] |
| Peptic ulcer disease | 0.63 [0.54; 0.75] |  | 0.79 [0.54; 1.16] |  | 0.92 [0.69; 1.21] |
| Liver disease | 0.91 [0.80; 1.04] |  | 0.58 [0.39; 0.88] |  | 1.09 [0.86; 1.38] |
| Renal disease | 0.67 [0.63; 0.71] |  | 0.54 [0.46; 0.63] |  | 1.40 [1.29; 1.52] |
| Malignancy | 0.68 [0.60; 0.76] |  | 0.55 [0.39; 0.76] |  | 1.12 [0.93; 1.35] |
| Diabetes | 1.07 [1.05; 1.10] |  | 0.91 [0.85; 0.98] |  | 1.32 [1.25; 1.39] |
| Hypertension | 1.00 [0.97; 1.02] |  | 0.81 [0.76; 0.86] |  | 0.95 [0.90; 0.99] |
| Dyslipidemia | 1.12 [1.09; 1.15] |  | 1.04 [0.97; 1.11] |  | 0.80 [0.75; 0.84] |
| Obesity | 1.21 [1.17; 1.25] |  | 0.80 [0.72; 0.89] |  | 0.85 [0.78; 0.92] |
| Smoking | 1.13 [1.09; 1.17] |  | 0.74 [0.66; 0.82] |  | 0.79 [0.73; 0.86] |

ACS: acute coronary syndrome; Afib/AFlutter: atrial fibrillation/atrial flutter; CABG: coronary artery bypass grafting; CHF: chronic heart failure; COPD: chronic obstructive pulmonary disease; CVD: cerebrovascular disease; ER: early readmission; NSTEMI: non-ST-segment elevation myocardial infarction; PCI: percutaneous coronary intervention; PVD: peripheral vascular disease; STEMI: segment elevation myocardial infarction; UA: unstable angina; Vfib/Vflutter: ventricular fibrillation/ventricular flutter
